# Dietary Supplementation with a Magnesium-Rich Marine Mineral Blend Enhances the Diversity of Gastrointestinal Microbiota

**DOI:** 10.3390/md16060216

**Published:** 2018-06-20

**Authors:** Erin K. Crowley, Caitriona M. Long-Smith, Amy Murphy, Elaine Patterson, Kiera Murphy, Denise M. O’Gorman, Catherine Stanton, Yvonne M. Nolan

**Affiliations:** 1 Department of Anatomy and Neuroscience, University College Cork, T12XF62 Cork, Ireland; erin.crowley@ucc.ie (E.K.C.); c.longsmith@ucc.ie (C.M.L.-S.); 2 APC Microbiome Ireland, Teagasc Food Research Centre, Moorepark, Fermoy, P61 C996 Cork, Ireland; amy.murphy@teagasc.ie (A.M.); elainepatterson01@gmail.com (E.P.); kieracmurphy@gmail.com (K.M.); catherine.stanton@teagasc.ie (C.S.); 3 School of Microbiology, University College Cork, T12YT20 Cork, Ireland; 4 Marigot Ltd., Strand Farm, Currabinny, Carrigaline, P43NN62 Cork, Ireland; denise.ogorman@marigot.ie; 5 APC Microbiome Ireland, University College Cork, T12YT20 Cork, Ireland

**Keywords:** marine extract, calcium, magnesium, gut microbial diversity, rat

## Abstract

Accumulating evidence demonstrates that dietary supplementation with functional food ingredients play a role in systemic and brain health as well as in healthy ageing. Conversely, deficiencies in calcium and magnesium as a result of the increasing prevalence of a high fat/high sugar “Western diet” have been associated with health problems such as obesity, inflammatory bowel diseases, and cardiovascular diseases, as well as metabolic, immune, and psychiatric disorders. It is now recognized that modulating the diversity of gut microbiota, the population of intestinal bacteria, through dietary intervention can significantly impact upon gut health as well as systemic and brain health. In the current study, we show that supplementation with a seaweed and seawater-derived functional food ingredient rich in bioactive calcium and magnesium (0.1% supplementation) as well as 70 other trace elements, significantly enhanced the gut microbial diversity in adult male rats. Given the significant impact of gut microbiota on health, these results position this marine multi-mineral blend (MMB) as a promising digestive-health promoting functional food ingredient.

## 1. Introduction

The increasing prevalence of a high fat/high sugar “Western diet” has been linked to the emergence of multiple health problems such as obesity, inflammatory bowel disease (IBD) [1], as well as cardiovascular [2,3,4], and metabolic diseases [5,6]. Prolonged exposure to this diet has also been associated with multiple vitamin and mineral deficiencies, including magnesium and calcium [7,8]. Across both Europe [9] and the U.S. [10], it has been repeatedly shown that adults are not meeting the recommended daily intake levels of both of these minerals, and more specifically in Ireland, the older population have been demonstrated to be particularly at risk [11]. Deficiencies in magnesium and calcium have been linked with an increased risk of malnutrition, cardiovascular disease [7,12], type 2 diabetes [13], decreased bone mineral density [14,15] and some cancers [16,17]. Conversely, mounting evidence suggest that dietary supplementation with these nutrients has a role to play in a healthy ageing process [8].

A Western diet has also been shown to markedly decrease the diversity of the gut microbiota, which is the population of intestinal bacteria that contribute to healthy gastro-intestinal functioning [18,19]. It also negatively impacts upon the immune system [20], the cardiovascular system [21], the brain, and behaviour [22]. There have been a considerable number of reports in recent years demonstrating that the composition of the microbiome can significantly impact not only an individual’s risk of systemic disease [22,23,24], but also the extent of disease progression [25,26,27,28]. It has been demonstrated that modulating the microbial composition may be a promising therapeutic avenue [29] for cancer [30], auto-immune [31,32], cardiovascular and kidney diseases [33,34]. Growing evidence suggests that modulating the diversity of the gut microbiota through dietary intervention (e.g., consuming a Mediterranean diet) can promote gut health and by extension systemic and brain health [35]. More specifically, several publications have postulated a link between the composition of the gut microbiota and the absorption of key minerals in the body including calcium and magnesium [36,37,38,39].

A seaweed-derived functional food ingredient, which is rich in bioactive calcium and magnesium, as well as 72 other trace minerals (Aquamin-F™), has previously been shown to have anti-inflammatory and other health benefits in terms of bone [40,41] and digestive health [42,43], as well as an ability to lower serum cholesterol levels in humans [44]. The potential benefits of a natural seawater-derived food ingredient (Aquamin-Mg^2+^™), which provides increased bioavailable magnesium levels for gut health has not yet been studied. The aim of this study was thus to investigate the effects of supplementation of these two functional food ingredients from marine sources (Marine Mineral Blend (MMB)), which in combination provide high levels of bioactive calcium and magnesium (see Table 1), on the diversity of the gut microbiota in adult rats.

## 2. Results

### 2.1. MMB Supplementation Had No Effect on Weight Gain, Food Intake or Locomotor Activity

**Figure 1:**
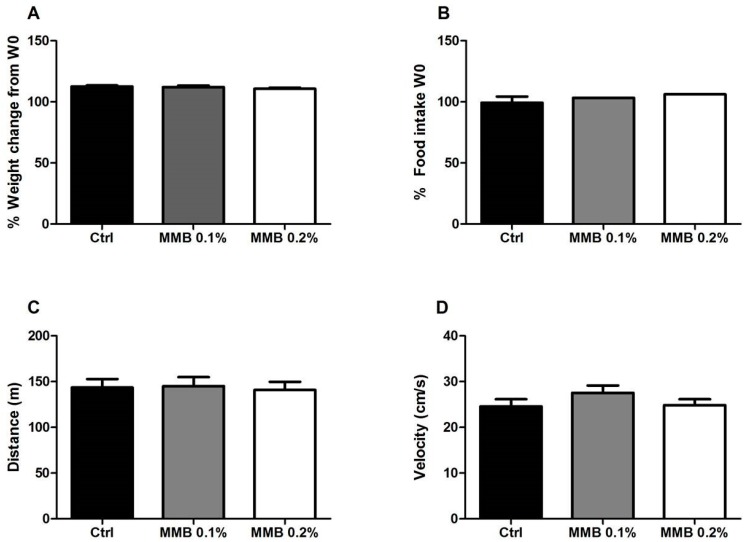
The effect of MMB supplementation on (**A**) weight gain and (**B**) food intake over the course of the experiment. General locomotor activity was measured in the open field using (**C**) distance travelled and (**D**) velocity travelled. Data are expressed as mean ± SEM (*n* = 10).

MMB-supplementation for six weeks did not significantly affect weight gain (F (2,27) = 0.96, *p* = 0.4; Figure 1A) or food intake (F (2,5) = 1.44, *p* = 0.36; Figure 1B) in rats when compared with body weight and food intake at the start of the study (week 0 (W 0)). MMB-supplementation had no effect on general locomotor activity in the open field, measured by either total distance (F (2,27) = 0.05, *p* = 0.95; Figure 1C) or velocity travelled (F (2,29) = 1.2, *p* = 0.31; Figure 1D).

### 2.2. MMB Supplementation Increased the Diversity of the Gut Microbiota

**Figure 2:**
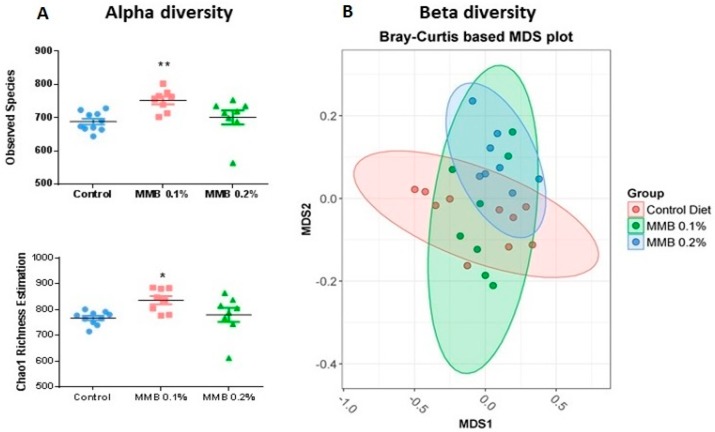
(**A**) Alpha diversity, quantified by the observed species and Chao1 richness estimation; and (**B**) beta diversity, represented by Bray-Curtis based multidimensional scaling (MDS) analysis of caecal microbiota composition in male Sprague Dawley rats fed a diet supplemented with 0.1% (green), 0.2% (blue) MMB or a standard rat chow (red) (*n *= 10) for 6 weeks.

There was a significant difference in the total number of observed species of bacteria measured by 16S rRNA gene sequencing between all groups (H (10.95), *p* = 0.004; Figure 2A). *Post hoc* analysis revealed that the MMB-0.1% supplemented group contained significantly more bacterial species than the control group (*p* < 0.01). The Chao1 index, which reflects species richness, also showed that there were significant differences between groups (H (8.1), *p* = 0.01; Figure 2A). *Post hoc* analysis revealed that this was caused by a significant increase in species in the MMB-0.1% supplemented group when compared to control (*p* < 0.05).

Permutational multivariate analysis of variance (PERMANOVA) analysis revealed a significant difference in beta-diversity between the groups (*p* = 0.014; Figure 2B). The principal component analysis (PCA) plot shows that the samples are separating based on MMB supplementation with data from the control group and both supplemented groups clustering together.

**Figure 3:**
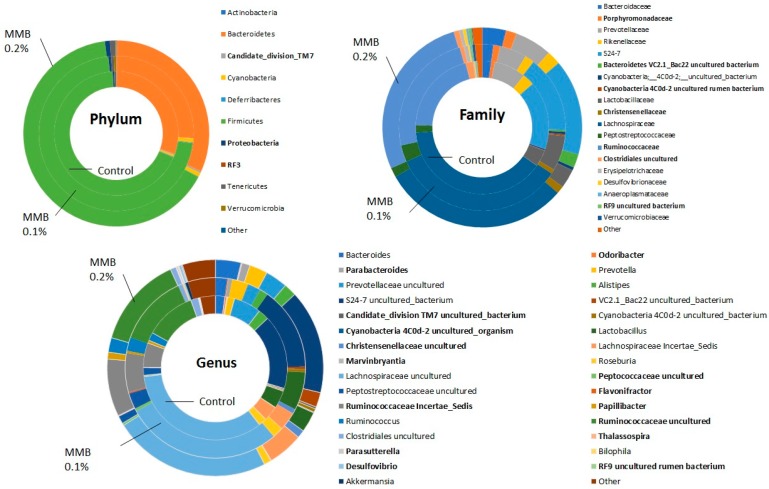
Relative abundance of caecal microbiota communities at the (**A**) Phylum level, (**B**) Family level, and (**C**) Genus level in male Sprague Dawley rats fed with a diet supplemented with 0.1%, 0.2% MMB or a standard rat chow (*n *=** 10) for 6 weeks. Only major taxonomic groups are shown. Significant differences are highlighted in bold. Statistical significance was accepted at *p* < 0.05. All *p* values were corrected for multiple comparisons using the Benjamini-Hochberg False Discovery Rate (pFDR).

Differences in the relative abundance between the groups were seen at all taxonomic levels. At the phylum level, there was a reduction in TM7 in the MMB-0.1% supplemented group when compared to both the control group and the MMB-0.2% supplemented group. Increased levels of Proteobacteria were also evident in rats in both supplemented groups. Additionally, there was a greater abundance of RF3 observed in rats from both supplemented groups when compared to rats in the control group. These differences at phylum level are reflected at lower taxonomic levels, as shown in Figure 3A,B. The significantly different taxa are shown in bold. This is particularly apparent at genus level with differences in several genera highlighted in Figure 3C.

### 2.3. MMB Supplementation Altered the Short Chain Fatty Acid (SCFA) Profile in the Gut

**Figure 4:**
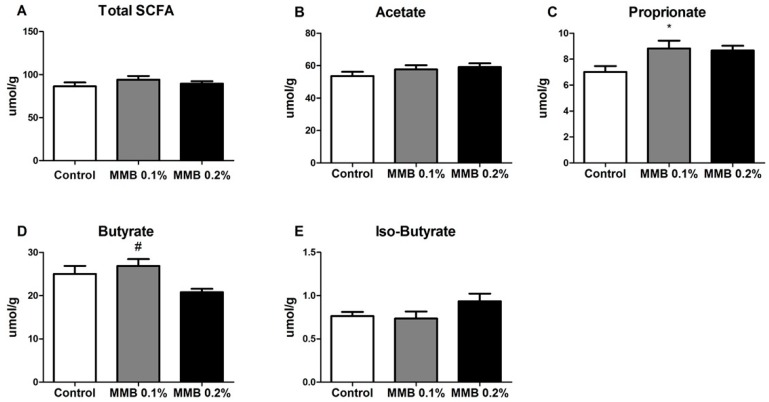
(**A**) Total short-chain fatty acid analysis, subdivided into (**B**) acetate; (**C**) propionate; (**D**) butyrate and (**E**) iso-butyrate. Data are expressed as mean ± SEM (*n* = 10). * *p* < 0.05 vs. control group, # *p* < 0.05 vs MMB-0.2% group.

There was no significant difference between the total number of SCFA present in caecal content in each group (F (2,29) = 0.97, *p* = 0.39; Figure 4A). MMB-supplementation had no effect on amounts of acetate (F (2,29) = 1.35, *p* = 0.28; Figure 4B) or iso-butyrate (F (2,29) = 2.15, *p* = 0.14; Figure 4E) presented. However, one-way analysis of variance (ANOVA) indicated that there was a statistically significant difference in amounts of propionate (F (2,29) = 4.36, *p* = 0.02; Figure 4C) and butyrate (F (2,29) = 4.46, *p* = 0.02; Figure 4D) presented between groups. *Post hoc* analysis revealed that this was caused by a significant increase in amount of propionate in the caecal content of MMB-0.1% supplemented rats compared to control (*p* < 0.05), and a significant increase in amount of butyrate in the caecal content of MMB-0.1% supplemented rats when compared to the MMB-0.2% supplemented group (*p* < 0.05).

## 3. Discussion

This study investigated the dose-dependent effects of dietary supplementation of a combination of a seaweed-derived and seawater-derived functional food ingredient with high levels of bioactive calcium and magnesium on gut microbiota composition in adult male rats. We found that incorporation of the supplement into the standard chow of rats resulted in an acceptable and palatable food product, as there were no differences in either the amount of food eaten or body weight gain between groups over the course of the experiment. Moreover, there was no impact on general activity as there were no differences in behaviour in the open field paradigm.

We report that six weeks of dietary supplementation with this MMB resulted in a significant increase in gut microbial diversity compared to control animals. Altered gut microbiota profiles and decreases in microbial diversity are evident in and may play a role in the pathophysiology of several immune and metabolic disorders [45] as well as in neurological and psychiatric disorders [22]. It should be noted, however, that an impaired biodiversity in the gastrointestinal microbiota is one of a myriad of health implications associated with these disorders, and it is not inconceivable that an increase in the bacterial spectrum may be associated with a detrimental health impact in certain circumstances. Several previous studies have shown that calcium supplementation results in increased gut microbial diversity in mice [46,47]. Moreover, magnesium-deficient diets have been shown to adversely affect gut microbial composition, and to promote both anxiety- and depressive-like behaviour in mice [48,49]. However, there is little evidence to date on the effects of magnesium supplementation on gut microbial diversity. In the present study, there were also significant changes observed in the gut microbiota composition at phylum, family and genus levels with many differences in relative abundance noted between the control group and both MMB supplemented groups. The phylum TM7 decreased from 0.29% of relative abundance in the caecal contents of the control group to 0.08% in the MMB-0.1% supplemented group, and increased to 0.31% in the MMB-0.2% supplemented group when compared with the MMB-0.1% supplemented group. This phylum has been associated with periodontitis, vaginosis and IBD [50]. Additionally, a high diversity of TM7 has been reported in patients with Crohn’s disease and IBD, and this phylum has been associated with mucosal inflammatory disease [51]. The relative abundance of Proteobacteria increased from 0.37% in the control group to 0.53% in the MMB-0.1% supplemented group, and significantly increased to 0.87% in the MMB-0.2% supplemented group. Proteobacteria have been linked to inflammation and metabolic syndrome and are usually found in low abundance in the gut of healthy individuals. Interestingly, it has been suggested that the increased prevalence of Proteobacteria is an indication of dysbiosis [52] and thus the constituent concentration of food or supplement ingredients that may potentially affect their abundance should be considered carefully. The phylum RF3 increased significantly between the control group and the MMB-0.2% supplemented group, however, it was present at very low levels (0.000034% to 0.011%). It is a candidate division, thus, no information is currently available on whether this phylum is beneficial or detrimental to gut health.

The level of Ruminococcaceae, which is one of the most abundant bacterial families in the mammalian gut and has been associated with the maintenance of gut health [53], was significantly increased in the caecal contents of MMB-0.2% supplemented group when compared to the control group. Specifically, Ruminococcaceae increased from 20% in relative abundance in the control group to 24% in the MMB-0.1% supplemented group and to 27% in the MMB-0.2% supplemented group. Members of this family are also involved in the degradation of indigestible carbohydrates which can then be converted to SCFAs [53]. An increase in the level of Christensenellaceae was also observed in the caecal contents of both of the MMB supplemented groups compared to the control group. This family increased 30-fold from the control group to the MMB-0.2% supplemented group (0.04% to 1.2%). It has been associated with a lean body mass index (BMI) and is enriched in the gut of healthy individuals. Members of this family have also been shown to promote a lean phenotype after microbiota transplant [54]. Additionally, a higher relative abundance of Porphyromonadaceae was observed in the MMB supplemented groups when compared to the control group. This family increased from 0.8% in the control group to 1.28% in the MMB-0.1% supplemented group, and then showed a further increase to 1.72% in the MMB-0.2% supplemented group. It has been suggested that members of the Porphyromonadaceae family are important for mediating gut health [55]. Furthermore, Porphyromonadaceae has been shown to have a protective effect on gut health after antibiotic treatment [56]. 

MMB-supplementation also resulted in a significant difference in the composition of SCFAs present in the gut of rats supplemented with 0.1% MMB, specifically leading to an increase in the concentration of butyrate and propionate. SCFAs are produced as a result of fermentation of carbohydrates by the resident bacterial microbiota [57,58], and so they can be directly linked to the changes in microbial diversity and composition. Growing evidence points to SCFAs having a wide variety of beneficial functions within the host, including working as signaling molecules and sources of energy as well as affecting immune function, lipid metabolism and gut integrity [59]. More specifically, butyrate and propionate have been shown to exert potent anti-inflammatory effects on the gut as well as decreasing oxidative stress and carcinogenesis [60,61,62]. Interestingly, this is in line with previous reports demonstrating anti-inflammatory effects of Aquamin-F^TM^ in both lipopolysaccharide-stimulated, glial-enriched cultures of cortex and in macrophages [63,64]. Conversely, decreased levels of intestinal SCFA have been directly correlated with a high-fat, high-sugar diet and associated altered gut microbial profile as well as with gut micro-inflammation [65]. Butyrate-producing species in the gut include Ruminococcaecae and Clostridaceae [61], both of which were significantly increased as a result of MMB supplementation.

For humans, the European Food Safety Authority (EFSA) advises an adequate intake for magnesium to be 300 mg/day for females and 350 mg/day for males (EFSA Panel on Dietetic Products and Allergies, 2015), while the recommended daily allowance (RDA) set by the US Institute of Medicine is 310–320 mg/day for females and 400–420 mg/day for males (Institute of Medicine, 2006). By these standards, the dose of magnesium used in the current study is relatively high. Indeed, high levels of magnesium have been shown to cause reversible osmotic diarrhea in humans [66]. We observed occasional incidents of diarrhea in rats on the 0.2% MMB supplement in the current study, thus this may be a reason why the 0.2% MMB supplemented diet did not show the same microbial compositional changes as the lower dose. Notwithstanding, in this proof-of-concept study in rats, the doses of magnesium which would have good potential bioavailability were selected based on a previous pioneering study which showed increased brain bioavailability of magnesium without any adverse effects. Indeed, enhanced cognitive changes were reported in the rats in this study [67]. Moreover, a recent review has highlighted that the solubility, dose and food matrix influence the availability of magnesium [68]. We cannot confirm that magnesium alone however is responsible for the observed effects in the current study due to its combination with >70 other trace natural minerals. It has been reported that when a mineral is supplemented in isolation, it is incapable of providing the same benefits as when that mineral is supplemented in concert with other complementary minerals [69]. This suggests that the marine magnesium component of MMB is enhanced by the full marine mineral profile in this functional food ingredient. We have shown that MMB-0.1% is tolerated well by rats and that it enhanced the microbial diversity, which can subsequently play a role in increased bioavailability through metabolic interactions [70]. Future research will determine the bioavailability of MMB relative to other magnesium salts and supplements.

In conclusion, we have shown that dietary supplementation with a seaweed and seawater-derived functional food ingredient rich in bioactive calcium and magnesium as well as more than 70 other trace elements, significantly enhanced the microbial diversity and composition of SCFAs in the gut of adult male rats. Given the significant effect of gut microbiota on systemic and brain health, these results position this marine mineral blend as a promising health promoting functional food ingredient. It should be noted, however, that research in this field is in its infancy. and there are as yet no definitive studies showing that a reversion of the narrowing of the microbial spectrum improves health outcomes in the long term. We propose that our current results contribute to the knowledge needed for the development of therapeutics for systemic diseases and will also establish a platform from which the effects of marine-derived compounds on systemic health can be investigated.

## 4. Materials and Methods

### 4.1. Dietary Supplementation and Experimental Design

Experimental chow was formulated by supplementing standard chow with a 50:50 mix of Aquamin F^TM^ (a mineral-rich food ingredient Aquamin^TM^, Marigot Ltd., Cork, Ireland, FDA GRAS000028; isolated from the red algae Lithoamnion species) and Aquamin-Mg^2+TM^ (enhanced with natural seawater-derived Mg^2+^) (Marigot Ltd., Cork, Ireland) to give either an additional 1 g magnesium/kg diet (Teklad Global custom diet, Envigo, Indianapolis, IN, USA), which equates to 50 mg magnesium/kg body weight per day; or an additional 2 g magnesium/kg diet, which equates to an additional 100 mg magnesium/kg body weight in an average 40 g rat. The lower-dose paradigm increased the total magnesium content × 1.5 fold, while the higher dose increased the magnesium content × 2 fold compared to the magnesium content in the standard chow. For simplicity, the term Marine Mineral Blend (MMB) is used here to indicate the experimental diets (see Table 1 for the complete mineral profile of MMB). The concentrations of magnesium formulated in MMB to be supplemented into the chow, were based on that used in a previous study which showed increased brain bioavailability of magnesium and associated cognitive functional changes without any adverse effects [67]. All of the mineral concentrations in the chow were approved by a nutritionist for safe consumption by rats (Envigo, USA).

Adult male Sprague Dawley rats (approximately 7–8 weeks of age, Envigo, Ely, UK) were maintained on a 12 h:12 h light: dark cycle (lights on at 08.00 h) under regulated temperature (21 ± 2 °C) and humidity (30–50%), with food and water available *ad libitum*. Animals were randomly divided into three groups and group housed with access to either standard rat chow (*n* = 10), 0.1% MMB-supplemented chow (*n* = 10) or 0.2%-supplemented MMB (*n* = 10). Animals were allowed free access to either control or MMB diets for 6 weeks before assessment of general locomotor activity in the open field. Body weight and food intake were recorded on a weekly basis for the duration of the study. Food intake was recorded as that in the last week of the experiment expressed as a percentage of total food intake of the first week of the experiment. The caecal content was collected at sacrifice after 6 weeks of dietary supplementation. All experiments were conducted in accordance with the European Directive 2010/63/EU, under an authorisation issued by the Healthcare Products Regulatory Authority Ireland (HPRA, AE19130/P021) and approved by the Animal Ethics Committee of University College Cork.

### 4.2. Open Field

Animals were placed in a white, round arena (90 cm in diameter) and their behaviour was recorded for 10 min. Motor activity including distance and velocity travelled were measured using Ethovision XT software (Version 11, Noldus, Leesburg, VA, USA). The apparatus was cleaned with 70% ethanol between each test to remove odour cues. One week after the open field test was carried out, animals were culled by decapitation. Caecal contents were snap frozen and stored at −80 °C until processing.

### 4.3. DNA Extraction

DNA extraction was performed as previously described [71]. DNA was extracted using the QIAmp Fast DNA Stool Mini Kit (Qiagen Ltd., Manchester, UK) according to manufacturer’s instructions, with the addition of an initial 3 min vortex step using 2 ml screw-cap tubes (Sarstedt, Wexford, Ireland) containing 0.25 g of a 1:1 mix of 0.1 mm and 1.5 mm diameter sterile zirconia beads plus a single 2.5 mm diameter bead (BioSpec Products, Bartlesville, OK, USA). Briefly, 200 mg of each caecal sample was added to a screw-cap tube containing beads with 1 ml of Qiagen InhibitEX^®^ Buffer (Qiagen, Manchester, UK) and vortexed for 3 min. Samples were incubated at 70 °C for 5 min for cell lysis and then centrifuged. Pelleted DNA was treated with proteinase K, the DNA was washed with buffers AW1 and AW2, and eluted in 200 μL Buffer ATE. DNA was quantified using the Qubit™ 3.0 Fluorometer (Bio-Sciences, Dublin, Ireland) along with the high sensitivity DNA quantification assay kit (Bio-Sciences, Dublin, Ireland).

4.4. 16S Compositional Sequencing

The V3-V4 region of the 16S rRNA gene was amplified and prepared for sequencing according to the 16S Metagenomic Sequencing Library Protocol (https://www.illumina.com/content/dam/illumina-support/documents/documentation/chemistry_documentation/16s/16s-metagenomic-library-prep-guide-15044223-b.pdf).

The protocol involved two PCR reactions on the extracted DNA. Firstly, the DNA was amplified using primers specific to the V3-V4 regions of the 16S rRNA gene: (forward primer 5′TCGTCGGCAGCGTCAGATGTGTATAAGAGACAGCCTACGGGNGGCWGCAG; reverse primer 5′GTCTCGTGGGCTCGGAGATGTGTATAAGAGACAGGACTACHVGGGTATCTAATCC). Each reaction contained 2.5 μL genomic DNA, 5 μL forward primer (1 μM), 5 μL reverse primer (1 μM) and 12.5 μL 2X Kapa HiFi Hotstart ReadyMix (Roche Diagnostics Ltd., West Sussex, UK). PCR amplification was carried out using the following program: 95 °C × 3 min, 25 cycles of 95 °C × 30 s, 55 °C × 30 s, 72 °C × 30 s, 72 °C × 5 min and held at 4 °C. PCR products were visualised using gel electrophoresis and then purified using AMPure XP beads (Labplan, Kildare, Ireland). Following this, a second PCR reaction was carried out on the purified DNA using two indexing primers per sample (Illumina Nextera XT indexing primers, Illumina Netherlands BV, Science Park, Eindhoven 5080, The Netherlands). Each reaction contained 5 μL purified DNA, 5 μL index 1 primer (N7xx), 5 μL index 2 primer (S5xx), 25 μL 2× Kapa HiFi Hot Start Ready mix (Roche Diagnostics, West Sussex, UK) and 10 μL PCR grade water. PCR amplification was completed using the previous program, with only eight amplification cycles instead of 25. PCR products were visualised and purified as described above. Samples were quantified using the Qubit™ 3.0 Fluorometer (Bio-Sciences, Dublin, Ireland) along with the high sensitivity DNA quantification assay kit and then pooled in an equimolar fashion (20 nM). The sample pool was prepared following Illumina guidelines and sequenced on the MiSeq sequencing platform in Clinical Microbiomics, Denmark using standard Illumina sequencing protocols.

### 4.5. Short-Chain Fatty Acid (SCFA) Analysis

SCFA analysis was performed as previously described [72] with some modifications. Approximately 0.2 g caecal content was mixed with 1.5 mL acidified Milli-Q water, this was vortexed well for 5–10 min and allowed to stand for 10 min at room temperature. The sample was then centrifuged at max speed for 5 min and the supernatant was filtered into fresh Eppendorfs using 0.2 μm filters. The filtered supernatant was then added to 10 mmol/L of internal standard (2-ethylbutyric acid (2-ETBA)). This was vortexed and centrifuged for 3 min before it was added to a fresh vial containing an insert. Samples were run in duplicate. Standard solutions containing 10.0, 8.0, 4.0, 2.0, 1.0, 0.5 and 0.1 mmol/L of a fatty acid mix containing iso-valeric acid, valeric acid, acetic acid, propionic acid, iso-butyric acid and butyric acid were used for calibration. The concentration of SCFA was measured using a Varian 3800 GC flame-ionisation system (Aquilant, Dublin 22, Ireland), fitted with a ZB-FFAP column (30 m × 0.32 mm × 0.25 μm; Phenomenex, Cheshire, UK). Helium was used as the carrier gas at a flow rate of 1.3 mL/min. The initial oven temperature was 100 °C for 0.5 min, raised to 180 °C at 8 °C/min and held for 1 min, then increased to 200 °C at 20 °C/min, and finally held at 200 °C for 5 min. The temperatures of the detector and injector were set at 250 °C and 240 °C, respectively. Peaks were integrated by using the Varian Star Chromatography Workstation version 6.0 software. Standards were included in each run to maintain the calibration.

### 4.6. Bioinformatic Analysis

Resulting 300 bp paired-end reads were assembled using FLASH (FLASH: fast length adjustment of short reads to improve genome assemblies; [73]). Further sequence read processing was performed using QIIME (Version 1.8.0) including quality filtering based on a quality score of >25 and removal of mismatched barcodes and sequences below length thresholds (QIIME allows analysis of high-throughput community sequencing data). Denoising, chimera detection and clustering into operational taxonomic units (OTUs) (97% identity) were performed using USEARCH (Version 7, 64-bit, [74], search and clustering orders of magnitude faster than BLAST). OTU sequences were aligned using PyNAST (PyNAST: python nearest alignment space termination; [75], a flexible tool for aligning sequences to a template alignment) and taxonomy was determined using the SILVA SSURef database release 111 [76] at 97% similarity. Alpha diversity estimates were calculated using QIIME. Beta diversity was calculated using Bray-Curtis based multidimensional scaling (MDS) analysis of caecal microbiota.

### 4.7. Statistical Analysis

All data were analyzed using either Statistica (Version 11, Statsoft, OK, USA) or R-3.3.1 ([77], accessed online) and graphed using GraphPad Prism (Version 5, La Jolla, CA, USA). Data are expressed as mean ± the standard error of the mean (SEM). Data were tested for normality, and statistical analysis was performed using one-way ANOVA for parametric data or Kruskal-Wallis for nonparametric data, followed by a post-hoc Dunn’s test if the ANOVA indicated significance. Differences were considered significant at *p* < 0.05. The vegan package was used for Bray-Curtis based MDS analysis, and the Adonis function in vegan was used for PERMANOVA in beta diversity. The Kruskal-Wallis test was used to identify significant differences between the groups, and the *p*-values were adjusted using the Benjamini-Hochberg method (FDR < 0.05).

## Figures and Tables

**Table 1:** Mineral composition of marine mineral blend. ppm, parts per million.

(>5 ppm)	ppm	(<5 ppm)	ppm	(<1 ppm)	(<0.2 ppm)
Calcium	285,000	Iodine	4.75	Hafnium	Rhodium
Magnesium	214,650	Barium	4.62	Cadmium	Tin
Carbon	106,500	Chromium	4.33	Antimony	Gallium
Sulfur	3174	Copper	3.73	Bismuth	Europium
Sodium	2835	Fluoride	2.97	Gold	Holmium
Chloride	1451	Zinc	2.77	Lithium	Terbium
Strontium	1262	Cerium	2.13	Selenium	Lutetium
Iron	975.50	Silver	2.07	Tellurium	Thulium
Silicon	380.00	Neodymium	1.92	Thallium	Rubidium
Aluminum	270.00	Lanthanum	1.66	Dysprosium	Tantalum
Manganese	265.35	Molybdenum	1.62	Praseodymium	Germanium
Potassium	142.60	Arsenic	1.47	Gadolinium	Cesium
Boron	110.80	Scandium	1.37	Erbium	Mercury
Phosphorus	96.65	Cobalt	1.24	Palladium	Platinum
Titanium	23.65	Nickel	1.19	Samarium	Iridium
Zirconium	10.45	Beryllium	1.10	Lead	Osmium
Vanadium	9.64	Ruthenium	1.10	Ytterbium	Rhenium
Thorium	9.08				Indium
Niobium	6.25				
Tungsten	5.57				
Yttrium	5.47				

## References

[1] Owczarek D., Rodacki T., Domagała-Rodacka R., Cibor D., Mach T. (2016). Diet and nutritional factors in inflammatory bowel diseases. World J. Gastroenterol..

[2] Nicoll R., Howard J.M.L., Henein M.Y. (2015). A review of the effect of diet on cardiovascular calcification. Int. J. Mol. Sci..

[3] Peterlik M., Cross H.S. (2005). Vitamin D and calcium deficits predispose for multiple chronic diseases. Eur. J. Clin. Investig..

[4] Vaskonen T. (2003). Dietary minerals and modification of cardiovascular risk factors. J. Nutr. Biochem..

[5] Moore-Schiltz L., Albert J.M., Singer M.E., Swain J., Nock N.L. (2015). Dietary intake of calcium and magnesium and the metabolic syndrome in the National Health and Nutrition Examination (NHANES) 2001–2010 data. Br. J. Nutr..

[6] Rayssiguier Y., Libako P., Nowacki W., Rock E. (2010). Magnesium deficiency and metabolic syndrome: Stress and inflammation may reflect calcium activation. Magnes. Res..

[7] Zhou B.F., Stamler J., Dennis B., Moag-Stahlberg A., Okuda N., Robertson C., Zhao L., Chan Q., Elliott P. (2003). Nutrient intakes of middle-aged men and women in China, Japan, United Kingdom, and United States in the late 1990s: TheINTERMAP Study. J. Hum. Hypertens..

[8] Ruxton C.H.S., Derbyshire E., Toribio-Mateas M. (2016). Role of fatty acids and micronutrients in healthy ageing: A systematic review of randomised controlled trials set in the context of European dietary surveys of older adults. J. Hum. Nutr. Diet..

[9] Burnett-Hartman A., Fitzpatrick A., Kun Gao M., Jackson S., Schreiner P. (2009). Supplement use contributes to meeting recommended dietary intakes for calcium, magnesium and vitamin C in four ethnicities of middle-aged and older Americans: The Multi-Ethnic Study of Atherosclerosis. J. Am. Diet. Assoc..

[10] Ford E.S., Mokdad A.H. (2003). Dietary Magnesium Intake in a National Sample of U.S. Adults. J. Nutr..

[11] Power S.E., Jeffery I.B., Ross R.P., Stanton C., O’Toole P.W., O’Connor E.M., Fitzgerald G.F. (2014). Food and nutrient intake of Irish community-dwelling elderly subjects: Who is at nutritional risk?. J. Nutr. Health Aging.

[12] Soderstrom L., Rosenblad A., Adolfsson E.T., Wolk A., Hakansson N., Bergkvist L. (2015). A high energy intake from dietary fat among middle-aged and older adults is associated with increased risk of malnutrition 10 years later. Br. J. Nutr..

[13] Fang X., Han H., Li M., Liang C., Fan Z., Aaseth J., He J., Montgomery S., Cao Y. (2016). Dose-response relationship between dietary magnesium intake and risk of type 2 diabetes mellitus: A systematic review and meta-regression analysis of prospective cohort studies. Nutrients.

[14] Ryder K.M., Shorr R.I., Bush A.J., Kritchevsky S.B., Harris T., Stone K., Cauley J., Tylavsky F.A. (2005). Magnesium intake from food and supplements is associated with bone mineral density in healthy older white subjects. J. Am. Geriatr. Soc..

[15] Rude R.K., Singer F.R., Gruber H.E. (2009). Skeletal and hormonal effects of magnesium deficiency. J. Am. Coll. Nutr..

[16] Ko H.J., Youn C.H., Kim H.M., Cho Y.J., Lee G.H., Lee W.K. (2014). Dietary magnesium intake and risk of cancer: A meta-analysis of epidemiologic studies. Nutr. Cancer.

[17] Chen G.C., Pang Z., Liu Q.F. (2012). Magnesium intake and risk of colorectal cancer: A meta-analysis of prospective studies. Eur. J. Clin. Nutr..

[18] Martinez K.B., Leone V., Chang E.B. (2017). Western diets, gut dysbiosis, and metabolic diseases: Are they linked?. Gut Microbes.

[19] Statovci D., Aguilera M., MacSharry J., Melgar S. (2017). The impact of western diet and nutrients on the microbiota and immune response at mucosal interfaces. Front. Immunol..

[20] El Aidy S., Dinan T.G., Cryan J.F. (2015). Gut Microbiota: The Conductor in the Orchestra of Immune-Neuroendocrine Communication. Clin. Ther..

[21] Marques F., Mackay C., Kaye D. (2018). Beyond gut feelings: How the gut microbiota regulates blood pressure. Nat. Rev. Cardiol..

[22] Sherwin E., Dinan T.G., Cryan J.F. (2017). Recent developments in understanding the role of the gut microbiota in brain health and disease. Ann. N. Y. Acad. Sci..

[23] Foster J.A., Rinaman L., Cryan J.F. (2017). Stress & the gut-brain axis: Regulation by the microbiome. Neurobiol. Stress.

[24] Ho J.T.K., Chan G.C.F., Li J.C.B. (2015). Systemic effects of gut microbiota and its relationship with disease and modulation. BMC Immunol..

[25] Neff C., Krueger O., Xiong K., Arif S., Nusbacher N., Schneider J.M., Cunningham A.W., Armstrong A., Li S., McCarter M.D. (2018). Faecal Microbiota Composition Drives Immune Activation in HIV-infected Individuals. EBioMedicine.

[26] Katsimichas T., Ohtani T., Motooka D., Tsukamoto Y., Kioka H., Nakamoto K., Konishi S., Chimura M., Sengoku K., Miyawaki H. (2018). Non-Ischemic Heart Failure With Reduced Ejection Fraction Is Associated With Altered Intestinal Microbiota. Circ. J..

[27] Li D., Tang W. (2018). Contributory Role of Gut Microbiota and Their Metabolites Toward Cardiovascular Complications in Chronic Kidney Disease. Semin. Nephrol..

[28] Zhou X., Li J., Guo J., Geng B., Ji W., Zhao Q., Li J., Liu X., Liu J., Guo Z. (2018). Gut-dependent microbial translocation induces inflammation and cardiovascular events after ST-elevation myocardial infarction. Microbiome.

[29] Feng Q., Chen W.D., Wang Y.D. (2018). Gut microbiota: An integral moderator in health and disease. Front. Microbiol..

[30] Goubet A.G., Daillère R., Routy B., Derosa L., Roberti P.M., Zitvogel L. (2018). The impact of the intestinal microbiota in therapeutic responses to cancer. Comptes Rendus-Biol..

[31] Perales-Puchalt A., Perez-Sanz J., Payne K.K., Svoronos N., Allegrezza M.J., Chaurio R.A., Anadon C., Calmette J., Biswas S., Mine J.A. (2018). Microbiota reconstitution restores intestinal integrity after cisplatin therapy. J. Leukoc. Biol..

[32] Opazo M.C., Ortega-Rocha E.M., Coronado-Arrázola I., Bonifaz L.C., Boudin H., Neunlist M., Bueno S.M., Kalergis A.M., Riedel C.A. (2018). Intestinal microbiota influences non-intestinal related autoimmune diseases. Front. Microbiol..

[33] Antza C., Stabouli S., Kotsis V. (2018). Gut microbiota in kidney disease and hypertension. Pharmacol. Res..

[34] Cosola C., Rocchetti M.T., Cupisti A., Gesualdo L. (2018). Microbiota metabolites: Pivotal players of cardiovascular damage in chronic kidney disease. Pharmacol. Res..

[35] Zoetendal E., de Vos W. (2014). Effect of diet on the intestinal microbiota and its activity. Curr. Opin. Gastroenterol..

[36] Weaver C.M. (2015). Diet, gut microbiome, and bone health. Curr. Osteoporos. Rep..

[37] Gomes J.M.G., Costa J.A., Alfenas R.C. (2015). Could the beneficial effects of dietary calcium on obesity and diabetes control be mediated by changes in intestinal microbiota and integrity?. Br. J. Nutr..

[38] Coudray C., Demigne C., Rayssiguier Y. (2003). Effects of Dietary Fibers on Magnesium Absorption in Animals and Humans. J. Nutr..

[39] Scholz-Ahrens K.E., Schaafsma G., Van den Heuvel E.G.H.M., Schrezenmeir J. (2001). Effects of prebiotics on mineral metabolism. Am. J. Clin. Nutr..

[40] Brennan O., Sweeney J., O’Meara B., Widaa A., Bonnier F., Byrne H.J., O’Gorman D.M., O’Brien F.J. (2017). A Natural, Calcium-Rich Marine Multi-mineral Complex Preserves Bone Structure, Composition and Strength in an Ovariectomised Rat Model of Osteoporosis. Calcif. Tissue Int..

[41] Brennan O., Stenson B., Widaa A., O’Gorman D.M., O’Brien F.J. (2015). Incorporation of the natural marine multi-mineral dietary supplement Aquamin enhances osteogenesis and improves the mechanical properties of a collagen-based bone graft substitute. J. Mech. Behav. Biomed. Mater..

[42] Aslam M., Paruchuri T., Bhagavathula N., Varani J. (2010). A Mineral-Rich Red Algae Extract Inhibits Polyp Formation and Inflammation in the Gastrointestinal Tract of Mice on a High-Fat Diet. Integr. Cancer Ther..

[43] Aviello G., Amu S., Saunders S.P., Fallon P.G. (2014). A mineral extract from red algae ameliorates chronic spontaneous colitis in IL-10 deficient mice in a mouse strain dependent manner. Phyther. Res..

[44] Cronin B.E., Allsopp P.J., Slevin M.M., Magee P.J., Livingstone M.B.E., Strain J.J., McSorley E.M. (2016). Effects of supplementation with a calcium-rich marine-derived multi-mineral supplement and short-chain fructo-oligosaccharides on serum lipids in postmenopausal women. Br. J. Nutr..

[45] Weiss G.A., Hennet T. (2017). Mechanisms and consequences of intestinal dysbiosis. Cell. Mol. Life Sci..

[46] Aslam M.N., Bassis C.M., Zhang L., Zaidi S., Varani J., Bergin I.L. (2016). Calcium reduces liver injury in mice on a high-fat diet: Alterations in microbial and bile acid profiles. PLoS ONE.

[47] Chaplin A., Parra P., Laraichi S., Serra F., Palou A. (2016). Calcium supplementation modulates gut microbiota in a prebiotic manner in dietary obese mice. Mol. Nutr. Food Res..

[48] Pyndt Jørgensen B., Winther G., Kihl P., Nielsen D.S., Wegener G., Hansen A.K., Sørensen D.B. (2015). Dietary magnesium deficiency affects gut microbiota and anxiety-like behaviour in C57BL/6N mice. Acta Neuropsychiatr..

[49] Winther G., Pyndt Jørgensen B.M., Elfving B., Nielsen D.S., Kihl P., Lund S., Sorensen D.B., Wegener G. (2015). Dietary magnesium deficiency alters gut microbiota and leads to depressive-like behaviour. Acta Neuropsychiatr..

[50] Dinis J.M., Barton D.E., Ghadiri J., Surendar D., Reddy K., Velasquez F., Chaffee C.L., Lee M.C.W., Gavrilova H., Ozuna H. (2011). In search of an uncultured human-associated TM7 bacterium in the environment. PLoS ONE.

[51] Kuehbacher T., Rehman A., Lepage P., Hellmig S., Fölsch U.R., Schreiber S., Ott S.J. (2008). Intestinal TM7 bacterial phylogenies in active inflammatory bowel disease. J. Med. Microbiol..

[52] Shin N.R., Whon T.W., Bae J.W. (2015). Proteobacteria: Microbial signature of dysbiosis in gut microbiota. Trends Biotechnol..

[53] Biddle A., Stewart L., Blanchard J., Leschine S. (2013). Untangling the genetic basis of fibrolytic specialization by Lachnospiraceae and Ruminococcaceae in diverse gut communities. Diversity.

[54] Goodrich J., Waters J., Poole A., Sutter J., Koren O., Blekhman R., Beaumont M., Van Treuren W., Knight R., Bell J. (2014). Human genetics shape the gut microbiome. PubMed Commons Cell.

[55] Zackular J.P., Baxter N.T., Iverson K.D., Sadler W.D., Petrosino J.F., Chen G.Y., Schloss P.D. (2013). The Gut Microbiome Modulates Colon Tumorigenesis. mBio.

[56] Ubeda C., Bucci V., Caballero S., Djukovic A., Toussaint N.C., Equinda M., Lipuma L., Ling L., Gobourne A., No D. (2013). Intestinal microbiota containing Barnesiella species cures vancomycin-resistant Enterococcus faecium colonization. Infect. Immun..

[57] Macfarlane G.T., Macfarlane S. (2011). Fermentation in the human large intestine: Its physiologic consequences and the potential contribution of prebiotics. J. Clin. Gastroenterol..

[58] Noble E.E., Hsu T.M., Kanoski S.E. (2017). Gut to Brain Dysbiosis: Mechanisms Linking Western Diet Consumption, the Microbiome, and Cognitive Impairment. Front. Behav. Neurosci..

[59] Morrison D.J., Preston T. (2016). Formation of short chain fatty acids by the gut microbiota and their impact on human metabolism. Gut Microbes.

[60] Hamer H.M., Jonkers D., Venema K., Vanhoutvin S., Troost F.J., Brummer R.J. (2008). Review article: The role of butyrate on colonic function. Aliment. Pharmacol. Ther..

[61] Louis P., Hold G.L., Flint H.J. (2014). The gut microbiota, bacterial metabolites and colorectal cancer. Nat. Rev. Microbiol..

[62] Louis P., Flint H.J. (2017). Formation of propionate and butyrate by the human colonic microbiota. Environ. Microbiol..

[63] Ryan S., Gorman D.M.O., Nolan Y.M. (2011). Evidence that the Marine-derived Multi-mineral Aquamin has Anti-inflammatory Effects on Cortical Glial-enriched Cultures. Phyther. Res..

[64] O’Gorman D.M., O’Carroll C., Carmody R.J. (2012). Evidence that marine-derived, multi-mineral, aquamin inhibits the NF-κB signaling pathway in vitro. Phyther. Res..

[65] Agus A., Denizot J., Thévenot J., Martinez-Medina M., Massier S., Sauvanet P., Bernalier-Donadille A., Denis S., Hofman P., Bonnet R. (2016). Western diet induces a shift in microbiota composition enhancing susceptibility to Adherent-Invasive *E. coli* infection and intestinal inflammation. Sci. Rep..

[66] Chassany O., Michaux A., Bergmann J. (2000). Drug-induced diarrhoea. Drug Saf..

[67] Slutsky I., Abumaria N., Wu L.J., Huang C., Zhang L., Li B., Zhao X., Govindarajan A., Zhao M.G., Zhuo M. (2010). Enhancement of Learning and Memory by Elevating Brain Magnesium. Neuron.

[68] Schuchardt J.P., Hahn A. (2017). Intestinal Absorption and Factors Influencing Bioavailability of Magnesium—An Update. Curr. Nutr. Food Sci..

[69] Strause L., Saltman P., Smith K., Bracker M., Andon M. (1994). Spinal Bone Loss in Postmenopausal Women Supplemented with Calcium and Trace Minerals. J. Nutr..

[70] Nicholson J.K., Holmes E., Kinross J., Burcelin R., Gibson G., Jia W., Pettersson S. (2012). Host-Gut Microbiota Metabolic Interactions. Science.

[71] Scott K.A., Ida M., Peterson V.L., Prenderville J.A., Moloney G.M., Izumo T., Murphy K., Murphy A., Ross R.P., Stanton C. (2017). Revisiting Metchnikoff: Age-related alterations in microbiota-gut-brain axis in the mouse. Brain Behav. Immun..

[72] Wall R., Marques T.M., O’Sullivan O., Ross R.P., Shanahan F., Quigley E.M., Dinan T.G., Kiely B., Fitzgerald G.F., Cotter P.D. (2012). Contrasting effects of Bifidobacterium breve NCIMB 702258 and Bifidobacterium breve DPC 6330 on the composition of murine brain fatty acids and gut microbiota. Am. J. Clin. Nutr..

[73] Magoč T., Salzberg S.L. (2011). FLASH: Fast length adjustment of short reads to improve genome assemblies. Bioinformatics.

[74] Edgar R.C. (2010). Search and clustering orders of magnitude faster than BLAST. Bioinformatics.

[75] Caporaso J.G., Bittinger K., Bushman F.D., Desantis T.Z., Andersen G.L., Knight R. (2010). PyNAST: A flexible tool for aligning sequences to a template alignment. Bioinformatics.

[76] Quast C., Pruesse E., Yilmaz P., Gerken J., Schweer T., Yarza P., Peplies J., Glöckner F.O. (2013). The SILVA ribosomal RNA gene database project: Improved data processing and web-based tools. Nucleic Acids Res..

[77] R-3.3.1 for Windows (32/64 bit). https://cran.r-project.org/bin/windows/base/old/3.3.1/.

